# Sitafloxacin- Versus Moxifloxacin-Based Sequential Treatment for Mycoplasma Genitalium Infections: Protocol for a Multicenter, Open-Label Randomized Controlled Trial

**DOI:** 10.2196/52565

**Published:** 2023-11-14

**Authors:** Naokatsu Ando, Daisuke Mizushima, Yosuke Shimizu, Yukari Uemura, Misao Takano, Morika Mitobe, Kai Kobayashi, Hiroaki Kubota, Hirofumi Miyake, Jun Suzuki, Kenji Sadamasu, Takato Nakamoto, Takahiro Aoki, Koji Watanabe, Shinichi Oka, Hiroyuki Gatanaga

**Affiliations:** 1 AIDS Clinical Center National Center for Global Health and Medicine Tokyo Japan; 2 Biostatistics Section, Department of Data Science National Center for Global Health and Medicine Tokyo Japan; 3 Department of Microbiology Tokyo Metropolitan Institute of Public Health Tokyo Japan

**Keywords:** bacteria, bacterial, clinical trial, clinical trials, controlled trials, doxycycline, drug, drugs, genital, genitalia, infection, medication, medications, moxifloxacin, Mycoplasma genitalium, pharmaceutic, pharmaceutical, pharmaceuticals, pharmaceutics, pharmacology, pharmacotherapy, pharmacy, quinolone resistance-associated mutation, randomized controlled trial, RCT, sexual transmission, sexually transmitted infection, sexually transmitted, sitafloxacin, STD, STI

## Abstract

**Background:**

*Mycoplasma genitalium* is an emerging sexually transmitted pathogen associated with increasing antibiotic resistance. The current treatment guidelines recommend moxifloxacin-sequential therapy for macrolide-resistant *M* *genitalium* or strains with unknown resistance profiles. However, it is unclear whether sitafloxacin, a 4th-generation fluoroquinolone antibiotic, is effective against resistant strains.

**Objective:**

This study aims to assess and compare the efficacy and safety of sitafloxacin- and moxifloxacin-based treatment regimens for managing *M* *genitalium* infections.

**Methods:**

We will conduct this randomized controlled trial at multiple centers in Japan. Eligible participants include adults aged 18 years or older with a confirmed *M* *genitalium* infection, as determined through the nucleic acid amplification test. Patients will be randomly assigned using a stratified approach based on the treatment facility and infection site. The interventions comprise oral sitafloxacin (200 mg) daily for 7 days (with optional pretreatment of oral doxycycline, 200 mg, daily for up to 7 days), with a control group receiving oral doxycycline (200 mg) daily for 7 days followed by moxifloxacin (400 mg) daily for another 7 days. The primary outcome is the treatment success rate with a superiority margin of 10%, as confirmed through the nucleic acid amplification test. Secondary outcomes encompass changes in the bacterial load at the urogenital or rectal sites and the emergence of posttreatment-resistant mutant strains.

**Results:**

Enrollment commenced in June 2023 and will conclude in December 2024, with findings anticipated by 2025. The expected success rates fall within the range of 80% for sitafloxacin and 42% for moxifloxacin against *M* *genitalium* carrying the G248T (S83I) mutation, based on previous studies. Accordingly, with a 5% significance level (2-sided) and 80% statistical power, we aim to recruit 50 participants per group, factoring in a 10% expected dropout rate.

**Conclusions:**

This study will provide valuable insights into the efficacy and safety of sitafloxacin- versus moxifloxacin-based sequential therapy in treating *M* *genitalium* infections. These findings have the potential to influence clinical guidelines, favoring more effective therapeutic choices. The multicenter approach enhances the robustness of this study. However, a limitation is the potential insufficiency of statistical power to detect posttreatment-resistant mutant strains in each group, rendering posttreatment-resistance mutations a notable concern. In the future, we may need to increase the sample size to enhance power.

**Trial Registration:**

Japan Registry of Clinical Trials (jRCTs031230111); https://jrct.niph.go.jp/en-latest-detail/jRCTs031230111

**International Registered Report Identifier (IRRID):**

DERR1-10.2196/52565

## Introduction

The increase in the number of resistant strains of *Mycoplasma genitalium* is a global concern. The global prevalence of *M genitalium* in the general population ranges from 1.3% to 3.9% [1]. The prevalence of macrolide resistance-associated mutations (MRMs) has increased over the decades [2]. Furthermore, regional variations in resistant strains have been observed, with the prevalence of MRMs in the American and Western Pacific regions exceeding that in European countries [2]. The prevalence of quinolone resistance-associated mutations (QRAMs) has been increasing in the Western Pacific regions, including Australia, New Zealand, China, and Japan, making these regions focal points for resistance mutations [3-7]. The current national guidelines recommend resistance-guided sequential therapy for *M genitalium* infections when macrolide resistance testing is available; otherwise, empiric sequential therapy is recommended, commencing with doxycycline followed by fluoroquinolone [8-11]. For macrolide-susceptible strains, sequential therapy involves an initial cycle of doxycycline treatment followed by azithromycin, whereas for macrolide-resistant strains, it entails an initial cycle of doxycycline treatment followed by fluoroquinolone. According to Australian studies, resistance-guided therapy with doxycycline followed by azithromycin attains a high efficacy of 92% to 95.4% against macrolide-susceptible strains, whereas doxycycline followed by moxifloxacin achieves a high efficacy of 95.4% against macrolide-resistant strains, and doxycycline followed by sitafloxacin demonstrates a high efficacy of 95% against macrolide-resistant strains [12-14]. However, the cure rate of fluoroquinolone-based therapies depends on the prevalence of QRAMs, which include *parC* and *gyrA* mutations. Specifically, S83 and D87 mutations in *parC*, as well as M95, G93, and D99 mutations in *gyrA*, have been linked to treatment failure with moxifloxacin and sitafloxacin [15-19]. Additionally, combined *parC* and *gyrA* mutations limit the efficacy of fluoroquinolone-based therapy more significantly than single mutations [19,20]. As sitafloxacin, a newer-generation fluoroquinolone, is not widely available, direct comparisons between moxifloxacin and sitafloxacin treatments are lacking. Sitafloxacin exhibits higher efficacy against QRAM-resistant strains [14,17,18] and demonstrates a lower minimum inhibitory concentration relative to moxifloxacin in QRAM-resistant strains based on minimum inhibitory concentration assays [17,18]. Murray et al [14] conducted a clinical study and demonstrated that sitafloxacin-based sequential therapy for the most common *parC* G248T (S83I) mutant strains achieved a higher success rate than moxifloxacin-based sequential therapy. However, as previously mentioned, they did not directly compare the 2 drugs but instead retrospectively compared them at different times. Given the fluctuating numbers of mutant-resistant strains in recent years, a randomized clinical trial would be a more suitable approach to investigate the efficacy of these 2 drug regimens [14,21].

In Japan, the prevalence of resistant strains is high [3,21]. Ando et al [3] reported that 89.6% of strains harbored MRMs, with 68.3% harboring *parC* mutations and 27% harboring *gyrA* mutations. Given the unavailability of resistance testing for MRMs and the high prevalence of strains harboring MRMs (89.6%), sitafloxacin is the preferred treatment for *M genitalium* infections at our facility. Ando et al [20] reported that sitafloxacin monotherapy cleared strains harboring wild-type *parC* and *gyrA*. In addition to resistant strains, sitafloxacin demonstrated high effectiveness (92.9%) in clearing strains harboring *parC* G248T (S83I) mutations and wild-type *gyrA*, with 41.7% of those harboring *parC* G248T (S83I) and *gyrA* mutations. In contrast, moxifloxacin-based therapy cleared 96.5% of the strains harboring wild-type *parC* and *gyrA* [19]. The regimens mainly comprised sequential therapy with doxycycline followed by moxifloxacin and combination therapy with doxycycline and moxifloxacin. However, for strains harboring *parC* G248T (S83I) mutations and wild-type *gyrA*, the effectiveness of moxifloxacin-based therapy was limited to 54.2%. For those harboring *parC* G248T (S83I) and *gyrA* mutations, the effectiveness was further reduced to 18.8% [19]. Both sitafloxacin and moxifloxacin are newer-generation quinolones that exhibit a comparable incidence of gastrointestinal side effects [22,23]. These agents are less frequently associated with tendon rupture than levofloxacin, a prevalent fluoroquinolone drug [24]. Considering that both are broad-spectrum antibiotics, their impact on de novo resistance and the environment remains unclear and requires vigilant monitoring. A 7-day course of doxycycline is generally used to treat sexually transmitted infections with mild side effects, such as gastrointestinal disturbances [25].

Accordingly, we propose to conduct a multicenter, open-label randomized controlled trial aimed at comparing the efficacy and safety profiles of sitafloxacin and standard moxifloxacin-based sequential treatments in managing highly resistant *M genitalium* infections.

## Methods

### Study Design

This study constitutes a comparative randomized open-label parallel-group trial assessing the efficacy of sitafloxacin- versus moxifloxacin-based sequential treatment for *M genitalium* infections, particularly those involving resistant strains.

### Population and Eligibility Criteria

**Figure 1:**
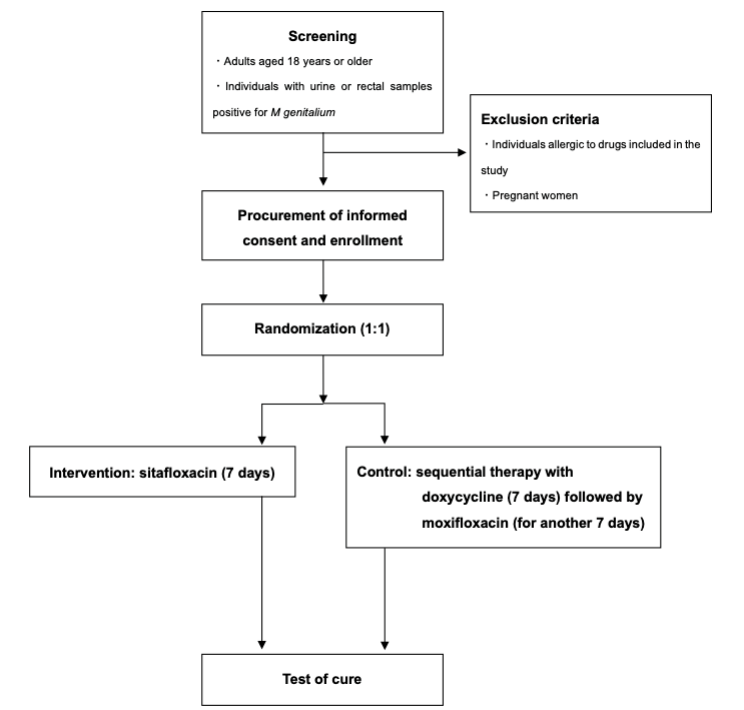
Study flow chart.

Participants are currently being recruited at the Sexual Health Clinic, AIDS Clinical Center of the National Center for Global Health and Medicine, and Personal Health Clinic in Japan (Figure 1). The inclusion criteria include several aspects. First, participants must be adults aged 18 years or older. Second, the confirmation of the presence of *M genitalium* in an individual’s urine or rectal samples is required through the nucleic acid amplification test (NAAT). Lastly, participants are obligated to provide written informed consent to partake in the study, with exclusion criteria covering individuals with known allergies to the study medications and pregnant women. Additionally, individuals deemed unsuitable for study participation by the attending physician based on various criteria or medical judgments will be excluded. Study participants who need antibiotics that are potentially effective for *M genitalium* infection. This includes quinolones other than the study drugs, as well as minocycline, pristinamycin, and nitroimidazole.

### Randomization and Blinding

We will use a computer-based stratified randomization approach to ensure an equitable allocation of participants across different treatment groups, stratifying based on 2 factors: the facility where the study participants are enrolled and the anatomical site of *M genitalium* infection (categorized as the urethra, rectum, or a combination of both). Participants will be randomly assigned to groups at a 1:1 ratio. Blinding will not be implemented, as treatment success will be determined using the NAAT, an objective test. After randomization, patients will receive their respective treatments and will be scheduled for a follow-up test of cure 3 weeks after completing the regimen, with a permissible range of 10-100 days. The test-of-cure content of the positive sites will be assessed using the NAAT. When treatment fails, the number of short tandem repeats in the protein MG309 will be analyzed to differentiate a new infection from a persistent one, as previously reported [26].

### Intervention

In the intervention group, participants will be administered 200 mg of oral sitafloxacin daily for 7 days. Additionally, 200 mg oral doxycycline daily for up to 7 days will be administered as pretreatment, depending on the severity, before the definitive diagnosis of *M genitalium*. Severity will be assessed based on patients’ complaints.

In the control group, participants will be initially administered 200 mg oral doxycycline daily for 7 days, followed by 400 mg moxifloxacin daily for another 7 days.

### Statistical Analysis

The sample size has been determined based on previous treatment success rates and specific assumptions. In a preliminary study, we determined that sitafloxacin achieved a treatment success rate of 92.9% against *M genitalium* with the *parC* G248T (S83I) mutation, in conjunction with wild-type *gyrA* [20]. Furthermore, sitafloxacin demonstrated a 78.9% success rate against strains carrying the *parC* G248T (S83I) mutation based on our preliminary data, and Murray et al [27] reported a moxifloxacin success rate of 41.2% against such strains. Accordingly, the success rates of sitafloxacin and moxifloxacin were anticipated to be 80% and 42%, respectively. With a 5% significance level (2-sided) and 80% statistical power for precise analysis, we aimed to enroll 50 participants per group (totaling 100 cases), considering the prevalence of the G248T mutation. Additionally, an expected dropout rate of approximately 10% was factored in, leading to a calculated sample size of 112 participants, evenly distributed across treatment groups, to robustly assess the efficacy of sitafloxacin and moxifloxacin against specific mutations in *M genitalium*.

Primary analyses regarding efficacy primarily rely on the full analysis set (FAS), which encompasses all registered participants, excluding ineligible participants and those not receiving the study drugs. To evaluate sensitivity, the analysis will be conducted for the per-protocol set, which consists of the study population, excluding cases of major protocol violations and discontinuations from the FAS. To evaluate safety, the analysis will be conducted for the safety analysis set, which includes all registered participants except those not receiving the study drug. Baseline characteristics of the study participants, both discrete and continuous, will be summarized. Discrete data will be presented as frequencies and percentages, whereas continuous data will be presented as descriptive statistics such as the mean and SD. Data will be analyzed with STATA (version 16.0; StataCorp), SAS software (version 9.4; SAS Institute), and R (version 4.1.2; R Foundation for Statistical Computing); the sample size calculation was done using Power Analysis and Sample Size 2022 (NCSS Statistical Software).

### Efficacy Outcome

The primary outcome is the treatment success rate, defined as the absence of the pathogen, as indicated through the NAAT. We set the superiority margin at 10%. The secondary outcomes include the change in bacterial load from pre- to posttreatment and the emergence of posttreatment nucleotide polymorphisms in mutant strains compared with those in pretreatment samples. The bacterial load will be measured as previously reported [28]. For the primary outcome in the FAS, with or without the G248T (S83I) mutation, rates and their 95% CIs will be computed. The chi-square test will be used to assess the statistical significance of treatment success between groups. This primary analysis will also be conducted for participants with the G248T mutation in the per-protocol set. In terms of secondary endpoints, differences, and rates of change in the bacterial load will be analyzed using either the 2-tailed Student *t* test or the Mann-Whitney *U* test. The rate and 95% CI of posttreatment resistance mutations in the FAS group will also be estimated using the positive cure confirmation results.

### Safety Outcomes

The side effects of each regimen will be reported during visit 2. Any unusual symptom or adverse reaction following the start of the regimens will be documented based on the division of the AIDS table for grading the severity of adult and pediatric adverse events (corrected version 2.1). Safety considerations include documenting adverse events, ranging from allergies and diarrhea to severe conditions such as aortic lesions, which are presented as frequency and group percentages for the safety analysis set.

### Data Collection and Management

A case report form (CRF) will be prepared by the appropriate and authorized individuals (investigators and physicians). All data recorded in the CRF must be consistent with the original material unless the data are recorded directly in the CRF as source material. Data will be collected at each visit (Table 1). All study findings and documents will remain strictly confidential, and patients will be identified only by their patient number or birth date, never by name, to protect their anonymity. The following data will be collected: date of birth (age), sex, nationality, race, medical history, history of current disease, complications, and allergies. Additionally, body weight and height will be measured at the first visit, along with vital signs at each visit, including the level of consciousness, body temperature (°C), blood pressure (mm Hg), pulse rate (beats per minute), respiratory rate, and physical condition at every visit, assessed through inspection, palpation, auscultation, and percussion.

**Table 1:** Study calendar.

Study activities	Screening	Visit 1 (day 1)	Visit 2 (day 28 or 35)^a^
Informed consent	✓		
Confirmation of eligibility (inclusion and exclusion)	✓		
Enrollment	✓		
Clinical characteristics	✓		
Physical findings	✓	✓	✓
Treatment		✓	
Adverse events			✓
Test of cure			✓

^a^Permissible range of 10-100 days.

### Data Monitoring

No external data monitoring committee will be involved in this study. The first author will oversee the progression, completion of the intervention, and follow-up assessment of all participants.

### Ethical Considerations

This trial was granted ethical approval by the National Center for Global Health and Medicine of Japan’s certified review board (approval NCGM-C-004624-00) in October 2022, registered under jRCTs031230111. The study protocol and informed consent forms were approved by the local ethics committees at each study site before the commencement of the study. Adherence to human subject ethics review approvals was maintained in accordance with institutional guidelines. Informed consent was obtained from all study participants, with the process including clear communication about their right to opt out at any stage without penalty. The data collected during the trial were anonymized to ensure the privacy and confidentiality of participants. While no monetary compensation was provided, participants received the study drug and associated testing free of charge. This provision was documented and justified according to ethical standards and guidelines, ensuring voluntary participation and the integrity of the study.

## Results

Enrollment began on June 1, 2023, and will continue until December 31, 2025, followed by participant follow-up. The findings of the study are expected to be released in 2025.

## Discussion

### Summary

The primary objective of this randomized controlled study is to determine the comparative efficacy and safety of sitafloxacin and moxifloxacin-based sequential treatments in managing *M genitalium* infections, with the primary outcome focusing on the clearance of the pathogen as confirmed by NAAT tests and the secondary outcomes considering bacterial load changes and posttreatment development of resistant strains. Based on previous studies, the expected success rates fall within the range of 80% for sitafloxacin and 42% for moxifloxacin against *M genitalium* carrying the G248T (S83I) mutation. Therefore, sitafloxacin is expected to demonstrate superior efficacy over moxifloxacin-based sequential therapy. The prevalence of strains harboring the G248T (S83I) mutation has increased in certain regions such as Australia, New Zealand, China, and Japan over recent years, with growing concerns in more areas such as the United States [29,30]. Accordingly, the outcomes of this study are poised to have a pivotal role in shaping the evolving treatment landscape for these pathogens, particularly the resistant strains, potentially influencing clinical practice. The multicenter design of this study also enhances its credibility by minimizing bias and broadening the applicability of our findings to a wider population.

### Limitations

This study has some limitations. One notable concern is the limited power to detect de novo resistance following the completion of each regimen. Furthermore, our focus extends to urogenital and rectal infections. While previous studies have suggested reduced efficacy of azithromycin against rectal infections, no discernible difference in the effectiveness of sitafloxacin was observed [20,31]. The treatment of rectal infections remains a subject of debate; however, considering that rectal infections may serve as potential reservoirs, assessing their effectiveness at both sites is crucial. Third, determining the optimal treatment for strains with both *parC* and *gyrA* mutations remains challenging owing to limitations in currently available effective regimens [19,20]. Exploring other drugs, such as nitroimidazole, with different mechanisms should be considered [32]. Considering these considerations, future studies, potentially encompassing a broader range of comparative therapies and exploring combinations of various treatments, must be conducted to ensure the best possible outcomes for patients.

### Conclusions

As the prevalence of *M genitalium* strains, particularly those with the G248T (S83I) mutation, increases globally, current research is imperative in guiding treatment.

When our hypothesis that sitafloxacin demonstrates superior efficacy over moxifloxacin-based sequential therapy holds, the study will have significant implications, potentially compensating for the practice, especially for resistant strains.

## Abbreviations

CRF: case report form
FAS: full analysis set
MRM: macrolide resistance-associated mutation
NAAT: nucleic acid amplification test
QRAM: quinolone resistance-associated mutation

## Data Availability

Data sharing is not applicable to this article, as no data sets have been currently generated or analyzed in the study.
